# A *Novel* Systems Pharmacology Method to Investigate Molecular Mechanisms of *Scutellaria barbata* D. Don for Non-small Cell Lung Cancer

**DOI:** 10.3389/fphar.2018.01473

**Published:** 2018-12-17

**Authors:** Jianling Liu, Meng Jiang, Zhihua Li, Xia Zhang, XiaoGang Li, Yuanyuan Hao, Xing Su, Jinglin Zhu, Chunli Zheng, Wei Xiao, Yonghua Wang

**Affiliations:** ^1^Key Laboratory of Resource Biology and Biotechnology in Western China, Ministry of Education, School of Life Sciences, Northwest University, Xi’an, China; ^2^Pharmacology Department, School of Pharmacy, Shihezi University, Shihezi, China; ^3^State Key Laboratory of New-tech for Chinese Medicine Pharmaceutical Process, Jiangsu Kanion Parmaceutical, Co., Ltd., Lianyungang, China

**Keywords:** non-small cell lung cancer, *Scutellaria barbata* D. Don, systems pharmacology, the molecular mechanism, baicalein

## Abstract

Non-small cell lung cancer (NSCLC) is the most ordinary type of lung cancer which leads to 1/3 of all cancer deaths. At present, cytotoxic chemotherapy, surgical resection, radiation, and photodynamic therapy are the main strategies for NSCLC treatment. However, NSCLC is relatively resistant to the above therapeutic strategies, resulting in a rather low (20%) 5-year survival rate. Therefore, there is imperative to identify or develop efficient lead compounds for the treatment of NSCLC. Here, we report that the herb *Scutellaria barbata* D. Don (SBD) can effectively treat NSCLC by anti-inflammatory, promoting apoptosis, cell cycle arrest, and angiogenesis. In this work, we analyze the molecular mechanism of SBD for NSCLC treatment by applying the systems pharmacology strategy. This method combines pharmacokinetics analysis with pharmacodynamics evaluation to screen out the active compounds, predict the targets and assess the networks and pathways. Results show that 33 compounds were identified with potential anti-cancer effects. Utilizing these active compounds as probes, we predicted that 145 NSCLC related targets mainly involved four aspects: apoptosis, inflammation, cell cycle, and angiogenesis. And *in vitro* experiments were managed to evaluate the reliability of some vital active compounds and targets. Overall, a complete overview of the integrated systems pharmacology method provides a precise probe to elucidate the molecular mechanisms of SBD for NSCLC. Moreover, baicalein from SBD effectively inhibited tumor growth in an LLC tumor-bearing mice models, demonstrating the anti-tumor effects of SBD. Our findings further provided experimental evidence for the application in the treatment of NSCLC.

## Introduction

Non-small cell lung cancer (NSCLC) is one of the leading causes of cancer death worldwide (Jemal et al., 2003). Faced with palliative care, chemotherapy is one of the main methods, but may cause severe side-effects and often leads to multidrug resistance (Ho et al., 2007). Therefore, the future of NSCLC treatment depends on the exploration and development of more effective drugs. In recent years, a large number of therapies, such as platinum therapies still represent the most common first-line treatment for NSCLC, however, it’s still difficult to achieve the most ideal treatment effect.

Traditional Chinese medicines (TCMs) are effective to relieve complicated diseases in a multi-target/multi-component manner, which makes them unique among all traditional medicines (Qiu, 2015), and have been used to treat various human diseases for over 4,000 years (Tang et al., 2009). For instance, *Scutellaria barbata* D. Don (SBD), is a perennial herb which is natively distributed in northeast Asia. This herb is known as Ban-Zhi Lian in TCMs which has been used to inhibit inflammatory (Dai et al., 2013) and block tumor (Wang, 2012) growth. Although SBD has been proven to be dramatically efficient in curing NSCLC (Wang Q. et al., 2018), the fundamental molecular action mechanisms are still not systematically explored. The bioactive compounds, the potential targets and the related pathways of SBD remain unknown. With the advancement of analytical tools such as systems biology (Kitano, 2002), network biology (Barabási and Oltvai, 2004) and network pharmacology (Hopkins, 2008), the intricate and holistic mechanisms of TCMs may be elucidated in a fast and highly effective way.

Recently, as a novelty discipline, systems pharmacology provides a new manner that integrates pharmacology and systems biology pharmacology, provides a new approach to explore TCMs across multiple scales of complexity ranging from molecular and cellular levels to tissue and organism levels (Berger and Iyengar, 2009). Systems pharmacology contains pharmacokinetics (ADME properties of drugs) evaluation, target prediction as well as network analysis (Huang et al., 2014), which offers a platform for identifying multiple mechanisms of action of medicine. In our previous work, the systems pharmacology method has been successfully applied to uncover the underlying function mechanisms of TCM formulas for cancer, depression, and cardiovascular diseases treatment (Li et al., 2014; Zhang et al., 2014; Zheng et al., 2014).

**FIGURE 1:**
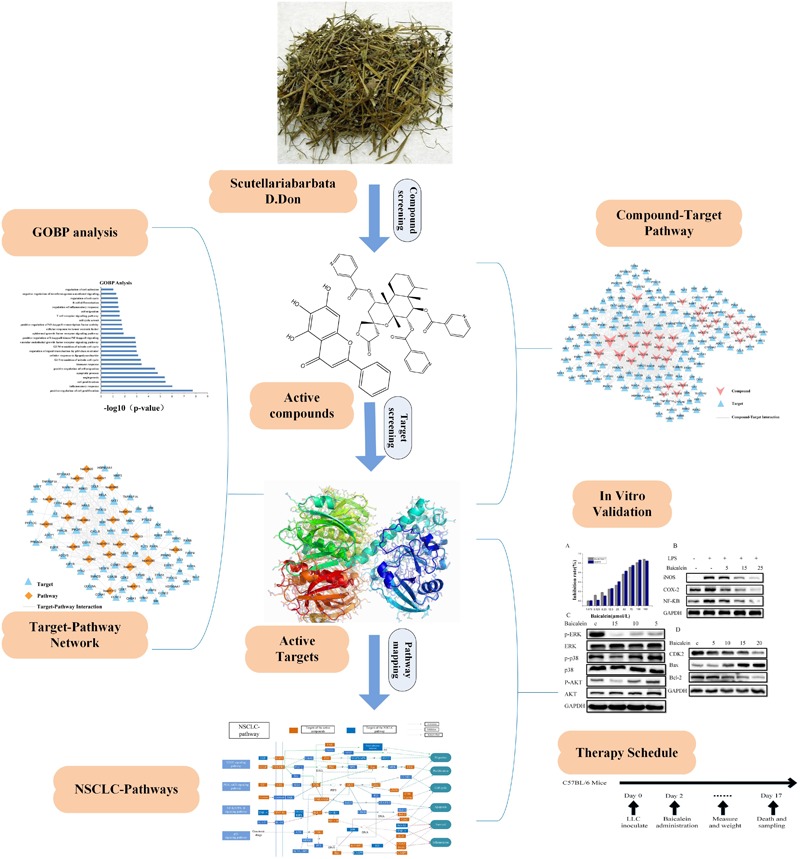
Systems pharmacology approach workflow.

Here, we introduce the method of systems pharmacology to resolve the underlying action mechanisms of herbal medicines in the treatment of NSCLC. Firstly, we filtered active compounds from the constructed SBD compound database by calculating pharmacokinetic properties and evaluating their oral bioavailability (OB) and drug-likeness (DL). Then, based on the integrated target prediction methods which united the biological and mathematical models, homologous targets of these active compound were predicted. Subsequently, obtained targets were validated by function enrichment analysis and target-disease interactions analysis. Ultimately, the network pharmacology and NSCLC-related signaling pathways evaluation were carried out to systematically disclose the underlying reciprocity between active compounds, active targets and pathways. The results not only significantly improved our understanding of NSCLC treatment mechanism, but also dissected the molecular mechanism of action of SBD, which promoted the exploitation of TCM in the treatment of sophisticated diseases. And *in vitro* experiments were conducted to evaluate the reliability of some vital active compounds and targets. Additionally, our *in vivo* results, which we subsequently confirmed using *in vitro* mechanism based assays, demonstrate that the significant anti-tumor activity of baicalein from SBD is associated with a direct impact of baicalein on improving tumor-inflammatory microenvironment. Our characterization of baicalein mediated changes in enzymes, cytokines, chemokines, and other growth factors associated with a tumor-inflammatory microenvironment offer multiple candidates to serve as potential biomarkers for ongoing clinical trials. In this paper, the detailed flow chart is shown in Figure 1.

## Materials and Methods

### Candidate Compound Database

All candidate compounds of SBD were manually collected from a wide scale text mining and our in-house developed database: the Traditional Chinese Medicine Systems Pharmacology Database (TCMSP^1^) (Ru et al., 2014). We got a total of 80 candidate compounds including flavonoids, terpenoids, and others. Glycosides were easy to hydrolyze into free glycosides absorbed by intestinal mucosa (Németh et al., 2003). Therefore, two aglycone compounds of glycosides in herbs were also added into the compound database for SBD. Eventually, we obtained 82 related compounds of SBD.

### ADME Screening

To investigate the active compounds of SBD that play a role in anti-NSCLC, we predicted the OB (predicted OB) and DL (predicted drug-likeness) values of them.

#### Oral Bioavailability

Oral bioavailability is considered to be an important indicator of the efficiency of active drug delivery to the systemic circulation, and OB is therefore one of the most important ADME of oral drugs. In this article, OB screening is calculated by a powerful internal system, OBioavail1.1 (Xu et al., 2012), we set the OB value mainly considering the following two factors as the basic principle. First, the information extracted from the studied medicines should be as much as probable with a minimum of molecules. Second, reasonably explaining the obtaining model by the reported pharmacological data (Wang et al., 2013). In this work, we have obtained an OB value of 30%, and the selected active compounds will be analyzed in the next step.

#### Drug-Likeness

Drug-likeness (DL) is used to estimate the similarity of physical properties of compounds with known drugs. In order to pick out the drug-like active molecules from SBD, based on molecular descriptors and Tanimoto similarity (Yamanishi et al., 2010; Liu et al., 2013), we used a self-constructed model DL to calculate the drug-likeness index of these compounds. The Drug-likeness evaluation method is as follows:

$$T(A,B)=(A*B\;)/({|A|}^{2}+{|B|}^{2\;}-A*B)$$

Here, A is defined as a molecular descriptor for herbal compounds and B is defined as the average molecular properties of all compounds in Drug Bank database^2^ (Wishart et al., 2008). In this work, a compound with DL ≥ 0.18 was selected as the active compound for further study.

In order to acquire the potential active compounds, the screening principle was defined as follows: OB ≥ 30%; DL ≥ 0.18.

### Target Prediction

To establish a direct link between the potential active compounds of SBD and the target, target selection for active compounds remains an urgent step. Therefore, compounds were further analyzed at the gene level. Firstly, targets exploration was fulfilled based on the weighted ensemble similarity (WES) and systematic drug targeting tool (SysDT). SysDT is a powerful computational model combining mathematics and bioinformatics. However, WES is a *in silico* model to pinpoint the drug direct targets of the actual bioactive ingredients (Liu et al., 2017). Secondly, we have mapped targets for UniProt^3^, unifying their names and organisms. Normalized compound targets are mapped to the CTD database^4^ (Davis et al., 2013), Therapeutic Target Database (TTD^5^) (Zhu et al., 2012), and Pharmacogenomics Knowledgebase (PharmGKB^6^) (Thorn et al., 2013) to obtain their associated diseases, providing a clearly defined target-disease relationship.

### GOBP

To probe the involved biological processes of the obtained targets, in this work, gene ontology (GO) enrichment analysis was performed by linking targets to DAVID (The Database for Annotation, Visualization and Integrated Discovery^7^) (Wang J. et al., 2018). Terms from “Biological Process” (GOBP) were utilized to symbol gene function (Yang et al., 2018). Only GO terms with *p*-value ≤ 0.05 were chosen. The false discovery rate (FDR) was introduced to reveal a multiple-hypothesis testing faulty measure of *p*-values by utilizing the web tool DAVID. We employed a 0.05 FDR criteria as an important cut off in our analysis.

### Network Construction

Currently, we have completed the screening and mapping of the active compounds and active targets. In order to investigate the multiple action mechanism of active compounds against NSCLC, and further clarify the relationship between active targets and active compounds. The Cytoscape 3.6.0 (Liu et al., 2016), a popular bioinformatics software package for biological network visualization and data integration was used. Two types of global networks were constructed: compound-target (C-T) network and target-pathway network (T-P) (Yu et al., 2012). In the magic network, compounds, targets, and pathways were represented by nodes, and the relationship between them was represented by the edges. In addition, degree (a vital topological parameter) was analyzed by the plug in Network Analyzer of Cytoscape (Shannon et al., 2003). The degree of a node is defined as the number of edges connected to that node.

In order to explore the integrative mechanisms of the formula for NSCLC, firstly, the activity target was mapped to the KEGG database^8^ (Kanehisa et al., 2017) and we got the basic information of the pathway. Secondly, according to the latest NSCLC pathological information, an integrated “NSCLC-Pathway” was assembled by integrating the key pathways that obtained through the T-P network and C-P network analysis.

### Experimental Validation

#### Cell and Mice

Human NSCLC H1975, RAW264.7 and Lewis lung carcinoma (LLC) cells were obtained from Chinese Academy of Sciences Shanghai cell bank. H1975 cells were cultured in RPMI1640 (Gibco) media complemented with 10% heat inactivated fetal bovine serum (FBS). RAW264.7 and LLC cells were cultured in DMEM (Gibco, United States) with 10% FBS. Cells were cultured at 37°C with 5% CO_2_ for all experiments. Mice were maintained under specific pathogen-free conditions at the Institute of Laboratory Animals, Jiangsu Kanion Parmaceutical, Co., Ltd. and used under protocols approved by the respective Institute of pharmacology and toxicology institutional review board (IRB), all animal experiments were performed in accordance with national and European guidelines. C57BL/6 mice (6–8 week-old) were purchased from the Comparative Medicine Centre of Yangzhou University. Female C57BL/6 wild type mice (6–8 week-old) were inoculated subcutaneously in the right flank with 5 × 10^5^ LLC cells per mouse (day 0). Before treatment, mice were then randomized into two groups: control (*n* = 6), Baicalein (*n* = 6). Baicalein (1.5 mg/kg, Yuan ye, Shanghai) was treated every day administration after the tumor inoculation (day 2). For untreated mice, an isotype control for physiological saline was intraperitoneally (i.p.) injected. Tumors were measured on every alternate day, and tumor volumes were calculated using the formula for a typical ellipsoid length × (width^2^) × 0.5(mm^3^). For survival decomposition, mice with tumors greater than the length limit of 20 mm were sacrificed and counted as dead. To examine the requirement of the priming and effector phases of tumor mass, the mice were sacrificed and tumors harvested for analysis, following 17 day observations and measurements.

#### Cell Viability Assay

Baicalein was purchased from Shanghai Yuanye Bio-Technology, Co., Ltd. (HPLC ≥ 98%, shanghai, China). Test samples were dissolved in dimethylsulfoxide (DMSO) (Sigma, United States) to get 100 mM, as a stock solution, and then stored at 4°C, it was not degrade due to high concentration of DMSO. The final dilutions of DMSO added to the culture medium never exceeded 0.1% what insured there was no effect on cell viability.

H1975 cells in the logarithmic phase were seeded at a density of 1 × 10^5^ cells/ml in 96-well culture plates. After incubated 48 h, cells were exposed to different concentrations of baicalein (1.675, 3.125, 6.25, 12.5, 25, 50, 100, and 150 μmol/L), RAW264.7 and H1975 cells have the same experimental protocol. After treatment for 48 h, then, 10 μl of CCK-8 assay (Best Bio, Shanghai, China) was added to each well and the cells were incubated for 1–4 h at 37°C and 5% CO_2_. A plate reader was used to detect the optical density (OD) absorbance at 450 nm. The cell viability was calculated as: OD of treatment/OD of control × 100%.

#### Western Blotting

Cells were scraped, collected by centrifugation and lysis in Qproteome^TM^ Mammalian Protein Prep Kit (Qiagen, Germany). The protein concentration of lysate was measured by a Quick Stari Bradford Protein Assay Kit (Bio-Rad, United States). Equal amount of protein taken from each sample was electrophoresis by 10% SDS-page gel electrophoresis and electroblotted onto nitrocellulose membranes, which were then incubated in a blocking buffer of 5% bovine serum albumin (BSA) in Tris-buffered saline. Primary antibody incubations were done overnight at 4°C in blocking buffer. After washing, secondary antibody incubations was done at room temperature for 1–1.5 h in blocking buffer. Primary antibodies recognizing the following proteins were obtained from ABcam: COX-2, iNOS, NF-κB, p38, ERK, p-p38, P-ERK, AKT, p-AKT, Bcl-2, CDK2, Bax, GAPDH. The membranes were detected by using the Clarity^TM^ Western ECL substrate (Bio-Rad) and labeling were visualized by Imagelab software (Bio-Rad).

#### Flow Cytometry Staining and Analysis

Tumors were digested with collagenase and hyaluronidase for 1 h at 37°C. After lysising of red blood cell, the dissociated cells were incubated on ice for 10 min, and then spun down at 300 g, 4°C for 7 min, cells from these tumors were either used for flow cytometry analysis or further processed and used for functional analyses. Tumor cell suspensions, were washed, blocked with Fc Block (anti-mouse CD16/32 mAb; BD Biosciences) at 4°C on ice for 15 min, and stained with fluorescence conjugated antibodies against surface markers CD49b, CD3E, CD8a, and CD25. These antibodies were purchased from BioLegend, eBioscience, or BD Biosciences. Cells were then fixed in Fixation/Permeabilization buffer (eBioscience) and stained with antibodies against intracellular proteins, including FoxP3 (BioLegend), granzyme B and interferon-γ (IFN-γ) (BD Pharmingen). Stained cells and isotype-control-stained cells, were assayed using a BD FACSVerse (BD Biosciences, United States). Data analysis was performed using the FlowJo (Tree Star) software.

#### Statistical Analysis

All Data are rendered as means ± standard error and the statistical results are analyzed by a one-way ANOVA and Student’s *t*-test. *p*-Values below 0.05 were considered as statistically significant.

## Results

### Active Compounds Screening

In this work, a total of 82 SBD related candidate compounds were collected from the SBD. To screen out the active compounds, it is significant to evaluate the compounds’ ADME properties including oral bioavailability (OB ≥ 30%) and drug-likeness (DL ≥ 0.18). As a result, based on the satisfactory screening conditions: 33 active components have been identified by us. In order to acquire more comprehensive results and make up for the theoretical screening deficiencies, some certain unqualified compounds, which have relatively poor pharmacokinetic properties, but are the most abundant and active compounds of certain herbs, were also selected as the active components for further study. For example, quercetin has poor OB (25%) property, it has been retained for further analysis as it is the main component of SBD and has anticancer and anti-inflammatory effects (Mukherjee and Khuda-Bukhsh, 2015). Also, luteolin with relatively poor OB (26.5%) was retained for further analysis since it exerts remarkable tumor suppressive activity on various types of cancers, including NSCLC (Jiang J et al., 2018). In the end, we obtained all 33 candidate components (Supplementary Table 1) (The structures of the compounds were derived from NCBI^9^) of SBD. Thereinto, flavonoids compounds have been reported to demonstrate significant biologic activity including anti-inflammatory, inhibit tumor angiogenesis, and cell cycle arrest (Zhang L. et al., 2017; Anwar et al., 2018). Such as, apigenin (MOL001 OB = 33.6% DL = 0.25), baicalein (MOL070 0B = 44.6% DL = 0.21). According to reports, diterpene compounds have activity test results, indicating that diterpene alkaloids have good cytotoxicity and can effectively inhibit the growth of a variety of human tumor cells (Lee and Ychoi, 2010), for example, Scutebarbatine F. In addition to the above components, SBD also contains ursolic acid, β-sitosterol, which has significant antitumor activity (Song et al., 2017; Rajavel et al., 2018). These active compounds could be the main elements for curing NSCLC.

### Target and Function Analysis

To get the targets related to NSCLC we firstly identified 225 targets of these active compounds by means of the WES and SysDT algorithms. The results shown that the candidate compounds act on multiple targets, and one target can also be linked to multiple candidate molecules. For example, target Nitric oxide synthase 2 (NOS2) corresponds to 15 compounds accounting for 45% of the total active compounds. Subsequently, as we know, increasing evidence has identified that improving the inflammatory microenvironment plays a crucial role in the research progress of NSCLC (Lee et al., 2017). Hence, the targets involved in the biological progress of NSCLC will be further preserved. Then, 225 candidate targets were mapping to the CTD, TTD, PharmGKB database to obtain the corresponding target related diseases. After screening, we finally retrieved 145 potential targets (Supplementary Table 2).

### GOBP Analysis

**FIGURE 2:**
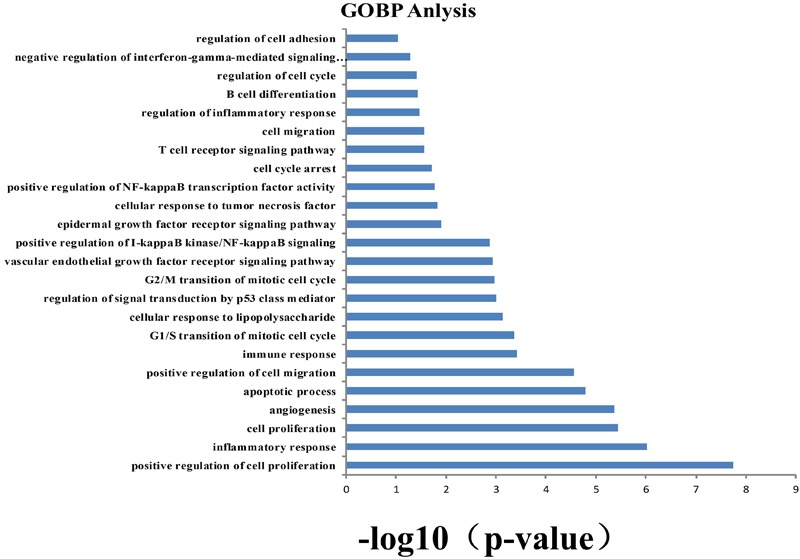
Gene ontology (GO) analysis of potential target genes. *y*-axis shows significantly enriched “biological process” (BP) categories in GO relative to the target genes, and *x*-axis shows the counts of targets.

To identify and analyze whether the biological process corresponding to the active target corresponds to NSCLC. GO (*p*-value ≤ 0.05) enrichment analysis was used to obtain 24 vital biological processes (Figure 2) by mapping targets to DAVID and screening. The results shown that the majority of these targets were strongly associated with various biological processes, including negative regulation of apoptosis process, positive regulation of cell proliferation, positive regulation of cell migration, inflammatory response, and angiogenesis. These biological processes are related to the research mechanism of NSCLC.

### Compound-Target Network Evaluation

**FIGURE 3:**
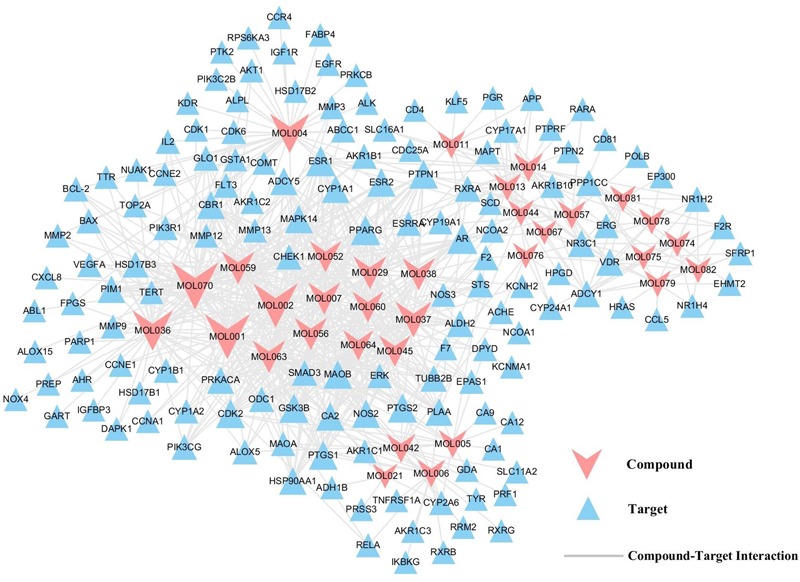
C-T network. A compound node and a target node are connected, if the gene is targeted by the consistent compound. Node size is relative to its degree.

In order to more directly reflect the relationship between targets and compounds, we have used Cytoscape 3.6.0 to map out the C-T relation network diagram. As shown in Figure 3, C-T diagram consist of 187 nodes (33 active compound nodes and 145 active target nodes) and 684 edges. Subsequently, C-T network topology analysis showed that the average degree of targets for each target was 4.7, illustrating the multi-target nature of SBD. Among the 33 active compounds, 25 of them show a high degree (degree > 10), which may play a key role in the network. Meanwhile, each active compound is associated with multiple targets, manifesting the potential synergistic effects among them.

Here, baicalein (MOL070) is the core component of SBD and display the highest number of target interactions (degree = 46). Previously, baicalein has been shown to have anti-cancer activities in several human cancer cell types including breast cancer, ovarian cancer, and colonic cancer (Yu et al., 2014; Wang et al., 2017; Dou et al., 2018). But beyond that, scutellarin (MOL036, degree = 39), a flavonoid used in Chinese herbal medicine, inhibited the proliferation and migration of human NSCLC cells (Sun et al., 2018). Also, wogonin (MOL007, degree = 27) is one of the active components of favonoids that are present in extracts from SBD. Recently, regulating immune function, anticancer and anti-inflammatory effects of wogonin have been discovered (Wang et al., 2010; Shi et al., 2017; Zhao et al., 2018). So, we speculate that the top three compounds might be the crucial elements in the treatment of NSCLC, which might exhibit anti-tumor and anti- inflammatory effects of SBD in the treatment of NSCLC. For instance, phosphatidylinositol-4,5-bisphosphate 3-kinase catalytic subunit gamma (PIK3CG, degree = 7) is targeted by seven active compounds from SBD. PIK3CG induces a transcription process that promotes immune suppression tumor growth and inflammation (Kaneda et al., 2017). Then, prostaglandin G/H synthase 2 (PTGS2, degree = 10) is simultaneously targeted by 10 active compounds, and has high expression in various tumors, which can promote tumor growth and regulates inflammatory response (Liu et al., 2018; Zhu et al., 2018). All of these suggest that SBD probably treat NSCLC by anti-inflammatory, inhibiting tumor angiogenesis, cell cycle arrest, and promoting apoptosis.

### Target-Pathway Network Evaluation

**FIGURE 4:**
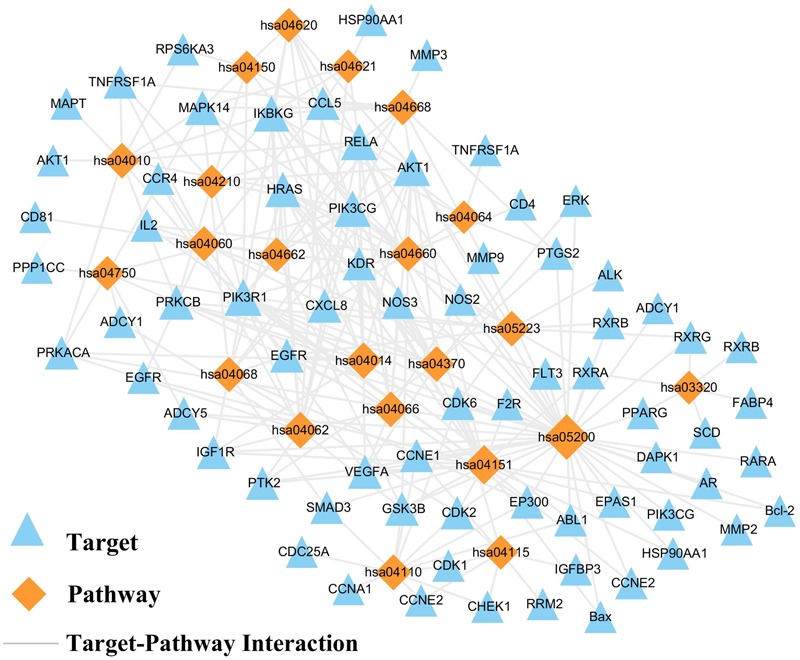
T-P network. The T-P network was consisted of targets and pathways, if the pathway is linked to the target. Node size is related to the degree.

The result displays that 145 targets are further mapped to 108 pathways, which show an average degree of 6.85 per pathway and 2.8 per target pathway. Then, we discover that several target proteins (71/145) are mapped to multiple pathways (≥5), exhibiting that these targets might intercede the interactions and cross-talk between different pathways. Meanwhile, numerous pathways (70/108), also regulated by multiple target proteins (≥8), might be the main factors for NSCLC. As shown in Figure 4, those pathways were tightly interacted with targets. Such as, PI3K-Akt signaling pathway (degree = 21), VEGF signaling pathway (degree = 11). For instance, The PI3K-Akt pathway is an important signaling pathway that may activate downstream of a series of extracellular signals and impact on cellular processes including cell proliferation, apoptosis, and survival (Jiang Z. Q. et al., 2018; Tan et al., 2018), which can be targeted by numerous active compound like wogonin (MOL007), baicalein (MOL070) quercetin (MOL002), apigenin (MOL001), and so forth. Hence, PI3K-Akt signaling pathway with the highest degree may be a significant pathway involved in the proliferation, apoptosis against NSCLC. Also, tumor angiogenesis, apoptosis, and migration related pathways were also enriched, like VEGF signaling pathway mediates the absolute dependence of tumor cells on the continuous supply of blood vessels to nourish their growth and to facilitate metastasis (Li et al., 2012). Hence, tumor vascularization is a vital process for tumor growth, invasion, and metastasis. SBD may be served as an attractive herb in anti-NSCLC therapy. In addition, P53 signaling pathway also stands out in the enriched pathway list, which can mediate cell cycle arrest and cell proliferation (Hsua et al., 2001). Therefore, it is concluded that SBD opportunity regulates the treatment of NSCLC through anti-inflammation, cell cycle arrest, tumor angiogenesis, cell apoptosis, and other pathways.

### NSCLC-Pathway Construction

**FIGURE 5:**
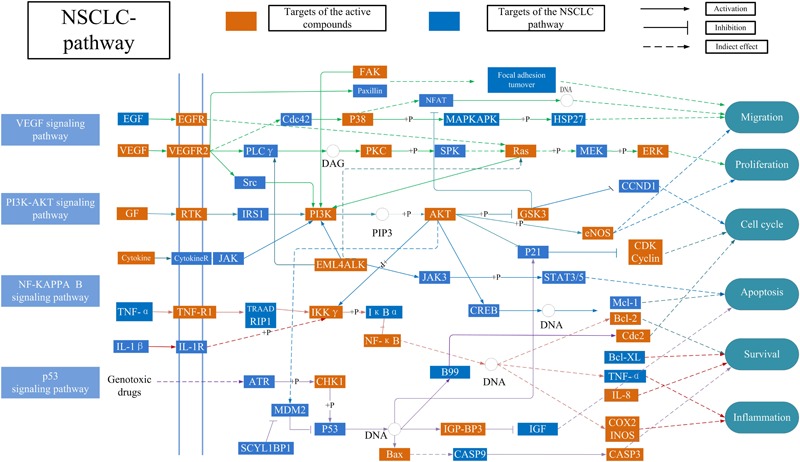
Distribution of target proteins of SBD on the compressed ‘NSCLC-pathway.’ Four pathways form the tubular NSCLC pathway. Arrows show activation active, T-arrows show inhibition active and segments represent indirectly activation effect or inhibition effect.

Considering the complex mechanism of SBD in the treatment of NSCLC, an integrated “NSCLC-pathway” was constructed by integrating the key pathways that obtained through the T-P network analysis. NSCLC-pathway (Figure 5) that comprises of four signaling pathways such as hsa04370: VEGF signaling pathway, hsa04151:PI3K-Akt signaling pathway, hsa04115: p53 signaling pathway and hsa04064: NF-kappa B signaling pathway. The target proteins of the integrated “NSCLC-pathway” exhibit markedly close functional relationship with the NSCLC related proteins. As shown in Figure 5, the NSCLC-pathway can be separated into two represent therapeutic modules (Inflammation related module and tumor related module). Inflammation related module consists of hsa04064: NF-kappa B signaling pathway. Then, tumor related module including hsa04370: VEGF signaling pathway, hsa04151: PI3K-Akt signaling pathway and hsa04115: p53 signaling pathway.

#### Inflammation Related Module

Inflammatory microenvironment plays a very important role in all stages of tumor development. Many important cytokines and chemical factors participate in this process. At the same time, the tumor microenvironment promotes the continuous response of the inflammation. Thus, it is necessary to control the development of the tumor by targeting the key signaling pathway in the tumor-inflammatory microenvironment. In Figure 5, baicalein (MOL070) activates transcription factor p65 (NF-κB), which reduces the expression of the downstream proteins prostaglandin G/H synthase 2 (COX-2) and Nitric oxide synthase (iNOS). For example, The NF-κB family of transcription factors is involved in the activation of a wide range of genes associated with inflammation, differentiation, tumorigenesis, embryonic development, and apoptosis (Ozawa et al., 2018; Zakaria et al., 2018). Then, COX-2 is not expressed in most normal tissues at high levels but is strongly induced by LPS and many cytokines, playing a crucial role in the development of various inflammatory responses (Zhang Q. et al., 2017). The results showed that baicalein form SBD could be utilized to treat NSCLC by regulating the anti-inflammatory activities of NF-κB, COX-2, and iNOS.

#### Tumor Related Module

As shown in the Figure 5, tumor related targets in the active targets are mapped to three pathways, including P53 signaling pathway, PI3K-AKT signaling pathway and VEGF signaling pathway. These pathways control tumor development by inhibiting cell proliferation and cell cycle. For example, in the P53 signaling pathway, baicalein (MOL070) can extensively act on cdk-Cyclin complexes and inhibit their activity, especially G1 phase cdk2-CyclinE (CDK2). It has been reported that the cell cycle is blocked by baicalein therapy, thus inhibiting cell proliferation (Hsua et al., 2001). These results suggested that baicalein from SBD inhibits NSCLC proliferation by incomplete DNA synthesis and cell division. Additionally, some targets in the PI3K-AKT signaling pathway engage in equaling the levels between the cell cycle and apoptosis. The apoptosis signaling can be initiated either at face through a death receptor-induced signaling pathway or within the cell via the release of proapoptotic molecules. For example, Apoptosis regulator Bcl-2 (Bcl-2) can be regulated by baicalein (MOL070), scutellarin (MOL036), and wogonin (MOL007). Previous data *in vivo* indicated that Caspases are linked to Bcl-2 family which is the key regulator of apoptosis in cancer (Timucin et al., 2018). Furthermore, tumor vascularization is an important process of tumor growth, invasion, and metastasis. Anti-angiogenesis has been considered to be an attractive target anti-tumor treatment (Ferrara and Kerbel, 2005; Cooney et al., 2006; Tran et al., 2007). Such as, In the VEGF signaling pathway was modulated by wogonin (MOL007) and baicalein (MOL070). It has been reported in the literature that inhibition of the activation of VEGF downstream protein kinases Mitogen-activated protein kinase 14 (p38) and Mitogen-activated protein kinase 3 (ERK) can inhibit cell proliferation, survival, and migration (Galaria et al., 2004). Thus, all above suggest that SBD may treat NSCLC by regulating the cell cycle, apoptosis, and anti-angiogenesis.

### *In vitro* Experimental Validation

#### CCK-8 Assay

**FIGURE 6:**
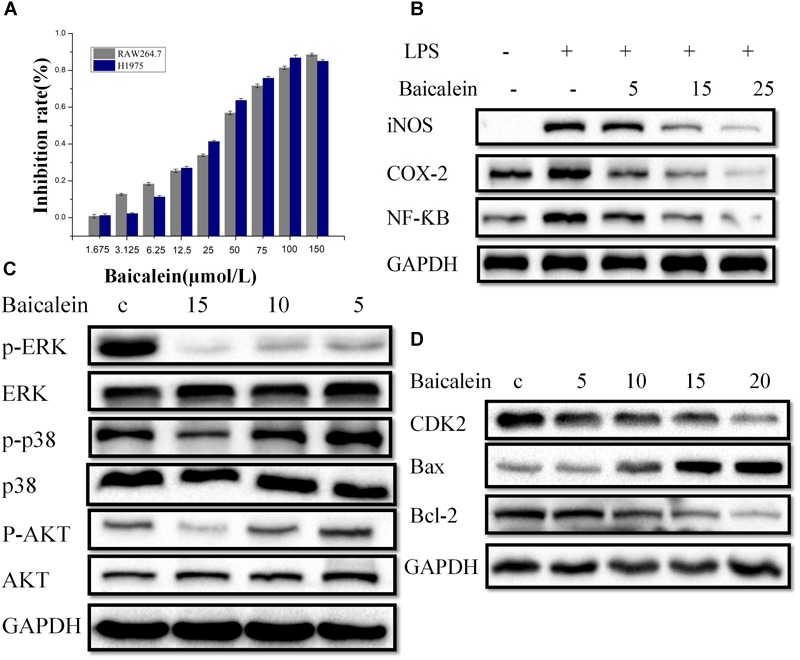
*In vitro* Experimental validation. **(A)** Cell viability of RAW264.7 and H1975 cells. The determination of cell viability of RAW264.7 and H1975 cells was carried out by CCK-8 assay after treated with control or baicalein for 48 h. **(B)** RAW264.7 cells were pretreated with (5, 15, or 25 μM), or the combinations for 2 h, vehicle as the control. The exposure to LPS (1 μg/ml) for 20 h, then iNOS, NF-κB and COX-2 accumulation of cytoplasm were measured by western blot. GAPDH was used as loading control. **(C)** H1975 cells were treated with baicalein (5, 10, or 15 μM) for 2 h, next, cell lysates were used to assess the phosphorylation of ERK, p38, and AKT. The total ERK, p38, and AKT are shown as a loading control. **(D)** Western blot analysis of CDK2, Bax, and Bcl-2 expression in H1975 cells. Cells were treatment with or without baicalein (5, 10, 15, or 20 μM) for 24 h. All results are repeated at least three independent experiments with the same tendency.

In our pre-experiment, the RAW 264.7 cell viability as affected by the baicalein from SBD at various doses was determined by CCK-8 assay (Figure 6A), and the outcome showed that high cell viability (>70%) was attained for baicalein at <25 μmol/L, respectively. Thus, three doses of baicalein were taken (5, 15, and 25 μmol/L) for subsequent experiments.

H1975 cells (1 × 10^5^ cells/ml) are cultured with the concentrations of 1.675 to 150 μmol per liter culture media with no serum of the baicalein for 48 h. Then, We performed CCK-8 assay to evaluate the inhibitory effects of baicalein induced proliferation of H1975 cells. Growth of H1975 cells was significantly inhibited by different concentrations of baicalein, and the 30% concentration of inhibition (IC30) of baicalein was 15 μmol/L respectively at 48 h (Figure 6A). Since lower doses baicalein had no inhibitory effect on the viability of H1975 cells, concentrations of baicalein (5, 10, 15, and 20 μmol/L) were chosen for subsequent experiments.

#### Targets Validation

To further assess the obtained results in systems pharmacology analysis, we have chosen baicalein from SBD to examine the compound potential anti-inflammatory effect using RAW264.7 cells treated with LPSs (1 ug/ml). In particular, we conduct western blot analysis for iNOS, NF-κB, and COX-2 protein expression to confirm anti-inflammatory effects of the predicted compounds.

As shown in Figure 6B, the levels of iNOS, NF-κB, and COX-2 proteins in the panel of RAW264.7 cell lines tested are reported. We observe that after baicalein treatment, the protein expressions of iNOS, NF-κB, and COX-2 in RAW264.7 cells are significantly declined. Figure 6B illustrates that baicalein treatment, as a single agent, causing a decrease of the iNOS, NF-κB, and COX-2 expression. To sum up, *in vitro* study provides additional information for screening compound with potentially anti-inflammatory effect and demonstrates the reliability of in systems pharmacology screen strategy.

To verify the reliability of anti-tumor related targets screened from systems pharmacology, we observe that baicalein treatment, the protein expressions of CDK2 and Bax in H1975 cells were both declined significantly at different dose levels. Our results show that after 24 h, an increase in Bax levels and a decrease in the level of CDK2 were detected in the P53 signaling pathway (Figure 6D), indicating that activating Bax and inhibiting CDK2 were vital for anti-NSCLC. Then, in the VEGF signaling pathway the phosphorylation of p38 was significantly down-regulated in baicalein-induced H1975 cells compared to those of the control group. Also, we examined the activation of ERK, baicalein inhibited phosphorylation of ERK in H1975 cells. However, total protein levels were not affected (Figure 6C), suggesting baicalein inhibited angiogenesis and cell proliferation by regulating VEGF signaling pathways. Next, we found that the p-AKT expression was down-regulated in H1975 cells treated with baicalein. In addition, the expression of Bcl-2 was decreased by baicalein treatment. The above results indicates that baicalein may suppress the PI3K/Akt pathway by down-regulating p-AKT (Figure 6C). Taken together, these data suggest that baicalein from SBD may treat the NSCLC by cell cycle arrest, promote apoptosis, and anti-angiogenesis.

### *In vivo* Experiments

**FIGURE 7:**
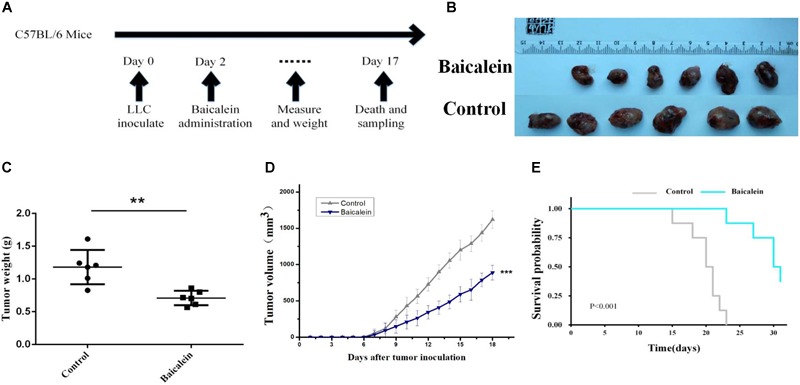
*In vivo* Experimental validation. **(A)** Schematic diagram of *in vivo* experimental schedule. **(B,C)** LLC-tumors derived from control and baicalein (5 × 10^5^ cells per C57B/L6 mice, six mice for each group) were photographed and weighed immediately after tumor extraction (mean ± SD; ^∗∗^*p* < 0.01). **(D,E)** LLC-bearing mice were treated with control and baicalein. Tumor volume and survival rates are shown in **(D,E)**. Data represent the means ± SEM of six mice. ^∗^*p* < 0.05, two-tailed Student’s *t*-test.

**FIGURE 8:**
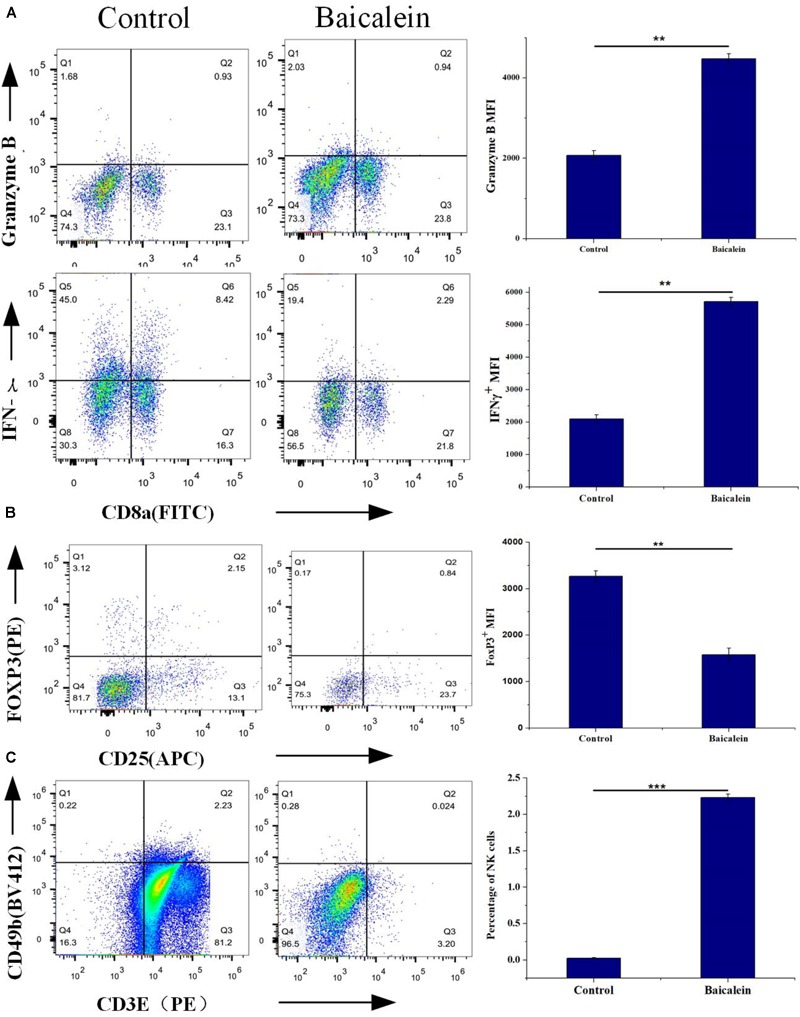
*In vivo* Experimental validation, tumor samples were isolated from LLC-bearing mice at the end of the study and processed for flow cytometry analysis. **(A)** Percentage of CD8+ Granzyme B+ and CD8+ IFNγ+ T cells of CD45+ cells were measured by Flow cytometry, and protein expression shown as mean fluorescence intensity (MFI). **(B)** Flow cytometry analysis of tumor samples shows the effect of control and baicalein treatment on Foxp3 levels. **(C)** FACS analysis of tumor-cell suspensions from LLC-bear mice after control or baicalein treatment. Quantification of NK cell present in the tumor samples. Results are shown as mean ± SEM (^∗∗^*p* < 0.01; ^∗∗∗^*p* < 0.001).

To elucidate the molecular mechanism of SBD in treating LLC tumor-bearing mice, we sought evidential insight for the driver of anti-tumor by improving the tumor-inflammatory microenvironment after baicalein from SBD administration (Figure 7A). LLC tumor-bearing mice were significantly more resistant to the development of baicalein mediate sarcoma than control mice and observed decreased tumor out growth (Figures 7B–D) and a significant survival benefit (Figure 7E) in baicalein-treated mice. Here, to evaluate the *in vivo* therapeutic effect of baicalein on NSCLC, tumor samples were collected from LLC tumor-bearing mice and subjected to fluorescence-activated cell sorting analysis. In the tumor-inflammatory microenvironment, we observed a significant increase in the proportion of cytotoxic CD8 + T cells in baicalein-treated mice, a sixfold of the proportion of CD8+/FoxP3+ cells compared to the control group. Also, reduction in the MFI of FoxP3+ Treg cells was observed in baicalein treatment group (Figure 8B), suggesting that an inhibition of Treg function associated with the reduction of FoxP3 protein expression (Ho et al., 2018) may be the direct result of the baicalein. Meanwhile, the baicalein therapy induced expansion of an IFNG and GZB-producing activated CD8+ T cell population in the tumor of mice (Figure 8A). Then, we examined the density of natural killer (NK) cells in the tumor sample. Significant positive correlations were observed between control and baicalein natural killer cells (Figure 8C). These results firmly establish that the baicalein from SBD therapy improves tumor inflammatory microenvironment mediated tumor control, resulting in a striking benefit in these advanced mouse models relevant to clinical.

## Discussion

Non-small cell lung cancer has a high degree of malignancy and it has the characteristics of an early metastasis, and the prognosis of NSCLC patients remains low despite the many advances that have occurred in early diagnosis and comprehensive therapies (Testa et al., 2018; Wang Z. et al., 2018). Therefore, search for antitumor drugs has become an important problem to be solved urgently.

Recently, SBD has been reported to possess vital biological activities, for instance anticancer activities (Zheng et al., 2018). In our work, the complex mechanism of SBD in the treatment of NSCLC was explored based on the system pharmacological work principle. Firstly, with the aid of the evaluation method, 33 active compounds were obtained from SBD and 145 active targets were predicted. These results reveal that the characteristics of SBD are multi-compounds and multi-targets anti-tumor effects. Then, target and C-T network analysis together display that some vital compounds of SBD such as wogonin, baicalein, and scutellarin may play an important role in the treatment of NSCLC, and SBD positively aiming for some targets like Bax, iNOS, and P38 exhibits the therapeutic effects against NSCLC by anti-inflammatory, promote apoptosis and anti-angiogenesis. In addition, The T-P network and the integrated NSCLC-relates pathway indicate that the major compounds of SBD might exert anti-NSCLC effect by modulating plenty different pathways including hsa04370:VEGF signaling pathway, hsa04151:PI3K-Akt signaling pathway, hsa04115:p53 signaling pathway and hsa04064:NF-kappa B signaling pathway. Based on our present study, the *in vitro* experiments further confirm that the baicalein from SBD combat NSCLC via regulating the critical proteins of our integrated NSCLC-pathway including COX-2, NF-κB, Bax, ERK, CDK1 and so on, attesting that NSCLC can be treated through a complex system with multi- compound-target-disease interactions. So, SBD exhibits anti-NSCLC effects in various aspects, including cell cycle arrest, anti-inflammatory, promoting apoptosis, and anti-angiogenesis in response to active compound. *In vivo*, additive therapeutic effects of baicalein were investigated for a tumor-bearing mouse model, where baicalein from SBD was demonstrated to possess high efficiency compared with control in the inhibition of tumor growth.

In summary, our study systematically indicated the inhibitory effect of SBD on anti-tumor *in vitro* and *in vivo*. These molecular mechanisms, including improve tumor inflammatory microenvironment, cell cycle arrest, promote apoptosis, and anti-angiogenesis are potentially those by which SBD exhibits its effectiveness in cancer treatment.

## Author Contributions

JL, MJ, and ZL contributed conception and design of the study. XZ and XL organized the database. JZ, YH, and XS performed the statistical analysis. MJ wrote the first draft of the manuscript. CZ, MJ, and WX wrote sections of the manuscript. All authors contributed to manuscript revision, read and approved the submitted version.

## Conflict of Interest Statement

The authors declare that the research was conducted in the absence of any commercial or financial relationships that could be construed as a potential conflict of interest.

## Footnotes

1 http://lsp.nwu.edu.cn/index.php

2 http://www.drugbank.ca/

3 http://www.uniprot.org

4 http://ctdbase.org/

5 http://database.idrb.cqu.edu.cn/TTD

6 https://www.pharmgkb.org

7 http://david.abcc.ncifcrf.gov

8 http://www.genome.jp/kegg/

9 https://pubchem.ncbi.nlm.nih.gov

## Abbreviations

ADME: absorption, distribution, metabolism, and excretion
LPSs: lipopolysaccharides
NSCLC: non-small cell lung cancer
SBD: *Scutellaria barbata* D. Don
TCMs: traditional Chinese medicines
