# Supplementary material for: A Novel Systems Pharmacology Method to Investigate Molecular Mechanisms of Scutellaria barbata D. Don for Non-small Cell Lung Cancer
Source: Front Pharmacol. 2018 Dec 17;9:1473. doi: 10.3389/fphar.2018.01473 (PMC6304355; doi:10.3389/fphar.2018.01473)
Supplement: Supplementary file 1 [file Data_Sheet_1.docx]

**Table 1**

Chemical information of 33 active compounds and their network parameters

| MOL_ID | Compounds | Structure | OB (%) | DL | Degree |
| --- | --- | --- | --- | --- | --- |
| MOL001 | luteolin |  | 26.5 | 0.25 | 43 |
| MOL002 | apigenin |  | 33.6 | 0.25 | 42 |
| MOL004 | quercetin |  | 25.0 | 0.77 | 37 |
| MOL005 | protocatechuic acid |  | 35.3 | 0.35 | 15 |
| MOL006 | vanillic acid |  | 60.4 | 0.40 | 15 |
| MOL007 | wogonin |  | 72.5 | 0.23 | 27 |
| MOL012 | beta-sitosterol |  | 36.5 | 0.75 | 14 |
| MOL013 | stigmasterol |  | 43.8 | 0.76 | 13 |
| MOL014 | ursolic Acid |  | 38.1 | 0.54 | 18 |
| MOL021 | isoscutellarein |  | 40.1 | 0.18 | 6 |
| MOL029 | dinatin |  | 71.6 | 0.27 | 29 |
| MOL036 | scutellarein |  | 36.6 | 0.78 | 39 |
| MOL037 | rivularin |  | 30.2 | 0.36 | 33 |
| MOL038 | salvigenin |  | 54.6 | 0.33 | 27 |
| MOL042 | 4-Methoxysalicylaldehyde |  | 31.4 | 0.32 | 17 |
| MOL044 | chlorogenic acid |  | 31.9 | 0.32 | 10 |
| MOL045 | moslosooflavone |  | 59.1 | 0.25 | 24 |
| MOL052 | baicalin |  | 67.5 | 0.24 | 24 |
| MOL056 | scutevulin |  | 36.0 | 0.26 | 27 |
| MOL057 | scutellaric Acid |  | 36.1 | 0.74 | 14 |
| MOL059 | 6-Hydroxyluteolin |  | 32.4 | 0.28 | 28 |
| MOL060 | (2S)-4',5,7-Trihydroxy-6-methoxyflavanone |  | 39.8 | 0.27 | 23 |
| MOL063 | naringenin |  | 69.2 | 0.21 | 28 |
| MOL064 | carthamidin |  | 54.5 | 0.24 | 21 |
| MOL067 | scutellarin |  | 30.2 | 0.70 | 4 |
| MOL070 | baicalein |  | 44.6 | 0.21 | 46 |
| MOL074 | scutebarbatine A |  | 34.6 | 0.78 | 9 |
| MOL076 | scutebarbatine E |  | 32.6 | 0.77 | 8 |
| MOL075 | scutebarbatine X |  | 31.4 | 0.68 | 7 |
| MOL078 | scutellone F |  | 37.6 | 0.74 | 9 |
| MOL079 | scutebarbatine I |  | 31.4 | 0.49 | 11 |
| MOL080 | scutebarbatine K |  | 36.3 | 0.78 | 9 |
| MOL082 | scutebarbatine J |  | 31.7 | 0.66 | 9 |

**Table 2**

The information of NSCLC-related targets of SBD

| UniProt ID | | Protein names | Gene | Degree | Sapiens |
| --- | --- | --- | --- | --- | --- |
| P00918 | Carbonic anhydrase 2 | | CA2 | 16 | *Homo sapiens* |
| P11309 | Serine/threonine-protein kinase pim-1 | | PIM1 | 13 | *Homo sapiens* |
| P10275 | Androgen receptor | | AR | 21 | *Homo sapiens* |
| P35228 | Nitric oxide synthase, inducible | | NOS2 | 15 | *Homo sapiens* |
| P37231 | Peroxisome proliferator-activated receptor gamma | | PPARG | 19 | *Homo sapiens* |
| P18031 | Tyrosine-protein phosphatase non-receptor type 1 | | PTPN1 | 17 | *Homo sapiens* |
| P23219 | Prostaglandin G/H synthase 1 | | PTGS1 | 17 | *Homo sapiens* |
| Q92731 | Estrogen receptor beta | | ESR2 | 16 | *Homo sapiens* |
| P49841 | Glycogen synthase kinase-3 beta | | GSK3B | 18 | *Homo sapiens* |
| P17612 | cAMP-dependent protein kinase catalytic subunit alpha | | PRKACA | 12 | *Homo sapiens* |
| P48736 | Phosphatidylinositol4,5-bisphosphate 3-kinase catalytic subunit gamma isoform | | PIK3CG | 7 | *Homo sapiens* |
| P24941 | Cyclin-dependent kinase 2 | | CDK2 | 17 | *Homo sapiens* |
| Q16539 | Mitogen-activated protein kinase 14 | | MAPK14 | 15 | *Homo sapiens* |
| P35354 | Prostaglandin G/H synthase 2 | | PTGS2 | 18 | *Homo sapiens* |
| P03372 | Estrogen receptor | | ESR1 | 20 | *Homo sapiens* |
| P07900 | Heat shock protein HSP 90-alpha | | HSP90AA1 | 13 | *Homo sapiens* |
| O95622 | Adenylate cyclase type 5 | | ADCY5 | 13 | *Homo sapiens* |
| O96020 | G1/S-specific cyclin-E2 | | CCNE2 | 2 | *Homo sapiens* |
| P04798 | Cytochrome P450 1A1 | | CYP1A1 | 13 | *Homo sapiens* |
| P05091 | Aldehyde dehydrogenase, mitochondrial | | ALDH2 | 11 | *Homo sapiens* |
| P05177 | Cytochrome P450 1A2 | | CYP1A2 | 4 | *Homo sapiens* |
| P08253 | 72 kDa type IV collagenase | | MMP2 | 3 | *Homo sapiens* |
| P08254 | Stromelysin-1 | | MMP3 | 3 | *Homo sapiens* |
| P09874 | Poly [ADP-ribose] polymerase 1 | | PARP1 | 2 | *Homo sapiens* |
| P09917 | Arachidonate 5-lipoxygenase | | ALOX5 | 14 | *Homo sapiens* |
| P14061 | Estradiol 17-beta-dehydrogenase 1 | | HSD17B1 | 1 | *Homo sapiens* |
| P14780 | Matrix metalloproteinase-9 | | MMP9 | 3 | *Homo sapiens* |
| P16152 | Carbonyl reductase [NADPH] 1 | | CBR1 | 14 | *Homo sapiens* |
| P21397 | Amine oxidase [flavin-containing] A | | MAOA | 4 | *Homo sapiens* |
| P24864 | G1/S-specific cyclin-E1 | | CCNE1 | 2 | *Homo sapiens* |
| P27338 | Amine oxidase [flavin-containing] B | | MAOB | 16 | *Homo sapiens* |
| P36888 | Receptor-type tyrosine-protein kinase FLT3 | | FLT3 | 5 | *Homo sapiens* |
| P37058 | Testosterone 17-beta-dehydrogenase 3 | | HSD17B3 | 8 | *Homo sapiens* |
| P39900 | Macrophage metalloelastase | | MMP12 | 2 | *Homo sapiens* |
| P45452 | Collagenase 3 | | MMP13 | 3 | *Homo sapiens* |
| P78396 | Cyclin-A1 | | CCNA1 | 2 | *Homo sapiens* |
| Q04760 | Lactoylglutathione lyase | | GLO1 | 6 | *Homo sapiens* |
| Q12882 | Dihydropyrimidine dehydrogenase[NADP(+)] | | DPYD | 3 | *Homo sapiens* |
| Q16678 | Cytochrome P450 1B1 | | CYP1B1 | 5 | *Homo sapiens* |
| Q9BVA1 | Tubulin beta-2B chain | | TUBB2B | 10 | *Homo sapiens* |
| Q9NPH5 | NADPH oxidase 4 | | NOX4 | 1 | *Homo sapiens* |
| Q9Y263 | Phospholipase A-2-activating protein | | PLAA | 13 | *Homo sapiens* |
| Q15596 | Nuclear receptor coactivator 2 | | NCOA2 | 4 | *Homo sapiens* |
| P29474 | Nitric oxide synthase, endothelial | | NOS3 | 3 | *Homo sapiens* |
| P22303 | Acetylcholinesterase | | ACHE | 3 | *Homo sapiens* |
| P00734 | Prothrombin | | F2 | 6 | *Homo sapiens* |
| P15090 | Fatty acid-binding protein, adipocyte | | FABP4 | 1 | *Homo sapiens* |
| Q13887 | Krueppel-like factor 5 | | KLF5 | 1 | *Homo sapiens* |
| P11388 | DNA topoisomerase 2-alpha | | TOP2A | 2 | *Homo sapiens* |
| O00750 | Phosphatidylinositol 4-phosphate 3-kinase C2 domain-containing subunit beta | | PIK3C2B | 1 | *Homo sapiens* |
| P05186 | Alkaline phosphatase, tissue-nonspecific isozyme | | ALPL | 1 | *Homo sapiens* |
| P05771 | Protein kinase C beta type | | PRKCB | 1 | *Homo sapiens* |
| P15121 | Aldose reductase | | AKR1B1 | 3 | *Homo sapiens* |
| P53985 | Monocarboxylate transporter 1 | | SLC16A1 | 3 | *Homo sapiens* |
| Q05932 | Folylpolyglutamate synthase, mitochondrial | | FPGS | 2 | *Homo sapiens* |
| O43570 | Carbonic anhydrase 12 | | CA12 | 1 | *Homo sapiens* |
| P00915 | Carbonic anhydrase 1 | | CA1 | 1 | *Homo sapiens* |
| P11509 | Cytochrome P450 2A6 | | CYP2A6 | 4 | *Homo sapiens* |
| P84022 | Mothers against decapentaplegic homolog 3 | | SMAD3 | 7 | *Homo sapiens* |
| Q16790 | Carbonic anhydrase 9 | | CA9 | 1 | *Homo sapiens* |
| Q9Y2T3 | Guanine deaminase | | GDA | 2 | *Homo sapiens* |
| P02766 | Transthyretin | | TTR | 2 | *Homo sapiens* |
| P42330 | Aldo-keto reductase family 1 member C3 | | AKR1C3 | 1 | *Homo sapiens* |
| Q04828 | Aldo-keto reductase family 1 member C1 | | AKR1C1 | 2 | *Homo sapiens* |
| P19793 | Retinoic acid receptor RXR-alpha | | RXRA | 8 | *Homo sapiens* |
| O14757 | Serine/threonine-protein kinase Chk1 | | CHEK1 | 7 | *Homo sapiens* |
| P11926 | Ornithine decarboxylase | | ODC1 | 6 | *Homo sapiens* |
| P27986 | Phosphatidylinositol 3-kinase regulatory subunit alpha | | PIK3R1 | 4 | *Homo sapiens* |
| P10636 | Microtubule-associated protein tau | | MAPT | 4 | *Homo sapiens* |
| P06401 | Progesterone receptor | | PGR | 2 | *Homo sapiens* |
| P04150 | Glucocorticoid receptor | | NR3C1 | 12 | *Homo sapiens* |
| P05067 | Amyloid-beta A4 protein | | APP | 3 | *Homo sapiens* |
| P05093 | Steroid 17-alpha-hydroxylase/17,20 lyase | | CYP17A1 | 5 | *Homo sapiens* |
| P06746 | DNA polymerase beta | | POLB | 1 | *Homo sapiens* |
| P11473 | Vitamin D3 receptor | | VDR | 12 | *Homo sapiens* |
| P36873 | Serine/threonine-protein phosphatase PP1-gamma catalytic subunit | | PPP1CC | 9 | *Homo sapiens* |
| Q08828 | Adenylate cyclase type 1 | | ADCY1 | 11 | *Homo sapiens* |
| Q07973 | 1,25-dihydroxyvitamin D(3) 24-hydroxylase, mitochondrial | | CYP24A1 | 3 | *Homo sapiens* |
| O60218 | Aldo-keto reductase family 1 member B10 | | AKR1B10 | 3 | *Homo sapiens* |
| P10276 | Retinoic acid receptor alpha | | RARA | 8 | *Homo sapiens* |
| P10586 | Receptor-type tyrosine-protein phosphatase F | | PTPRF | 1 | *Homo sapiens* |
| P11308 | Transcriptional regulator ERG | | ERG | 5 | *Homo sapiens* |
| P11511 | Aromatase | | CYP19A1 | 3 | *Homo sapiens* |
| P17706 | Tyrosine-protein phosphatase non-receptor type 2 | | PTPN2 | 1 | *Homo sapiens* |
| P55055 | Oxysterols receptor LXR-beta | | NR1H2 | 7 | *Homo sapiens* |
| P31350 | Ribonucleoside-diphosphate reductase subunit M2 | | RRM2 | 1 | *Homo sapiens* |
| P35030 | Trypsin-3 | | PRSS3 | 1 | *Homo sapiens* |
| P00325 | Alcohol dehydrogenase 1B | | ADH1B | 1 | *Homo sapiens* |
| Q15788 | Nuclear receptor coactivator 1 | | NCOA1 | 1 | *Homo sapiens* |
| P14222 | Perforin-1 | | PRF1 | 1 | *Homo sapiens* |
| Q12791 | Calcium-activated potassium channel subunit alpha-1 | | KCNMA1 | 1 | *Homo sapiens* |
| P08842 | Steryl-sulfatase | | STS | 6 | *Homo sapiens* |
| P11474 | Steroid hormone receptor ERR1 | | ESRRA | 4 | *Homo sapiens* |
| Q99814 | Endothelial PAS domain-containing protein 1 | | EPAS1 | 4 | *Homo sapiens* |
| P17936 | Insulin-like growth factor-binding protein 3 | | IGFBP3 | 1 | *Homo sapiens* |
| P22102 | Trifunctional purine biosynthetic protein adenosine-3 [Includes: Phosphoribosylamine--glycine ligase | | GART | 1 | *Homo sapiens* |
| P08709 | Coagulation factor VII | | F7 | 1 | *Homo sapiens* |
| Q12809 | Potassium voltage-gated channel subfamily H member 2 | | KCNH2 | 2 | *Homo sapiens* |
| P49281 | Natural resistance-associated macrophage protein 2 | | SLC11A2 | 1 | *Homo sapiens* |
| P14679 | Tyrosinase | | TYR | 1 | *Homo sapiens* |
| P15428 | 15-hydroxyprostaglandin dehydrogenase [NAD(+)] | | HPGD | 1 | *Homo sapiens* |
| Q96RI1 | Bile acid receptor | | NR1H4 | 3 | *Homo sapiens* |
| O00767 | Acyl-CoA desaturase | | SCD | 1 | *Homo sapiens* |
| P25116 | Proteinase-activated receptor 1 | | F2R | 6 | *Homo sapiens* |
| P33527 | Multidrug resistance-associated protein 1 | | ABCC1 | 4 | *Homo sapiens* |
| P37059 | Estradiol 17-beta-dehydrogenase 2 | | HSD17B2 | 1 | *Homo sapiens* |
| P06493 | Cyclin-dependent kinase 1 | | CDK1 | 2 | *Homo sapiens* |
| P10145 | Interleukin-8 | | CXCL8 | 1 | *Homo sapiens* |
| P16050 | Arachidonate 15-lipoxygenase | | ALOX15 | 1 | *Homo sapiens* |
| P48147 | Prolyl endopeptidase | | PREP | 1 | *Homo sapiens* |
| Q00534 | Cyclin-dependent kinase 6 | | CDK6 | 4 | *Homo sapiens* |
| Q96KQ7 | Histone-lysine N-methyltransferase EHMT2 | | EHMT2 | 2 | *Homo sapiens* |
| Q8N474 | Secreted frizzled-related protein 1 | | SFRP1 | 3 | *Homo sapiens* |
| P01112 | GTPase HRas | | HRAS | 1 | *Homo sapiens* |
| P13501 | C-C motif chemokine 5 | | CCL5 | 1 | *Homo sapiens* |
| Q09472 | Histone acetyltransferase p300 | | EP300 | 2 | *Homo sapiens* |
| P31749 | RAC-alpha serine/threonine-protein kinase, | | AKT1 | 1 | *Homo sapiens* |
| Q9UM73 | ALK tyrosine kinase receptor | | ALK | 1 | *Homo sapiens* |
| P51679 | C-C chemokine receptor type 4 | | CCR4 | 1 | *Homo sapiens* |
| P00533 | Epidermal growth factor receptor | | EGFR | 1 | *Homo sapiens* |
| P47116 | Serine/threonine-protein kinase PTK2/STK2 | | PTK2 | 1 | *Homo sapiens* |
| P08069 | Insulin-like growth factor 1 receptor | | IGF1R | 1 | *Homo sapiens* |
| P60568 | Interleukin-2, IL-2 | | IL2 | 4 | *Homo sapiens* |
| P51812 | Ribosomal protein S6 kinase alpha-3, S6K-alpha-3, | | RPS6KA3 | 1 | *Homo sapiens* |
| O60285 | NUAK family SNF1-like kinase 1 | | NUAK1 | 2 | *Homo sapiens* |
| P15692 | Vascular endothelial growth factor A | | VEGFA | 4 | *Homo sapiens* |
| P35968 | Vascular endothelial growth factor receptor 2 | | KDR | 1 | *Homo sapiens* |
| Q9Y6K9 | NF-kappa-B essential modulator | | IKBKG | 1 | *Homo sapiens* |
| P28702 | Retinoic acid receptor RXR-beta | | RXRB | 1 | *Homo sapiens* |
| P48443 | Retinoic acid receptor RXR-gamma | | RXRG | 1 | *Homo sapiens* |
| Q04206 | Transcription factor p65 | | RELA | 3 | *Homo sapiens* |
| P19438 | Tumor necrosis factor receptor superfamily member 1A | | TNFRSF1A | 1 | *Homo sapiens* |
| P01730 | T-cell surface glycoprotein CD4 | | CD4 | 1 | *Homo sapiens* |
| P30304 | M-phase inducer phosphatase 1 | | CDC25A | 1 | *Homo sapiens* |
| P60033 | CD81 antigen | | CD81 | 1 | *Homo sapiens* |
| P35869 | Aryl hydrocarbon receptor, Ah receptor, AhR | | AHR | 2 | *Homo sapiens* |
| P53355 | Death-associated protein kinase 1, DAP kinase 1 | | DAPK1 | 2 | *Homo sapiens* |
| O14746 | Telomerase reverse transcriptase | | TERT | 2 | *Homo sapiens* |
| P00519 | Tyrosine-protein kinase ABL1 | | ABL1 | 1 | *Homo sapiens* |
| P52895 | Aldo-keto reductase family 1 member C2 | | AKR1C2 | 1 | *Homo sapiens* |
| P21964 | Catechol O-methyltransferase | | COMT | 1 | *Homo sapiens* |
| P08263 | Glutathione S-transferase A1 | | GSTA1 | 1 | *Homo sapiens* |
| P27361 | Mitogen-activated protein kinase 3, MAP kinase 3, | | ERK | 4 | *Homo sapiens* |
| P10415 | Apoptosis regulator Bcl-2 | | BCL-2 | 5 | *Homo sapiens* |
| Q07812 | Apoptosis regulator BAX | | BAX | 5 | *Homo sapiens* |
